# OTP970 Is Required for RNA Editing of Chloroplast *ndhB* Transcripts in *Arabidopsis thaliana*

**DOI:** 10.3390/genes13010139

**Published:** 2022-01-14

**Authors:** Mei Fu, Xiaona Lin, Yining Zhou, Chunmei Zhang, Bing Liu, Dongru Feng, Jinfa Wang, Hongbin Wang, Honglei Jin

**Affiliations:** 1 School of Life Sciences, Sun Yat-sen University, Guangzhou 510275, China; meizi07166@126.com (M.F.); zhangcm@163.com (C.Z.); liubing@mail.sysu.edu.cn (B.L.); Issfdr@mail.sysu.edu.cn (D.F.); Isswjf@mail.sysu.edu.cn (J.W.); 2 School of Pharmaceutical Sciences, Institute of Medical Plant Physiology and Ecology, Guangzhou University of Chinese Medicine, Guangzhou 510006, China; linnxn@163.com (X.L.); zhou_yining@126.com (Y.Z.); wanghongbin@gzucm.edu.cn (H.W.)

**Keywords:** *Arabidopsis*, chloroplast, ndhB-C149, OTP970, PPR, RNA editing

## Abstract

RNA editing is essential for compensating for defects or mutations in haploid organelle genomes and is regulated by numerous *trans*-factors. Pentatricopeptide repeat (PPR) proteins are the prime factors that are involved in RNA editing; however, many have not yet been identified. Here, we screened the plastid-targeted PLS-DYW subfamily of PPR proteins belonging to *Arabidopsis thaliana* and identified ORGANELLE TRANSCRIPT PROCESSING 970 (OTP970) as a key player in RNA editing in plastids. A loss-of-function *otp970* mutant was impaired in RNA editing of *ndhB* transcripts at site 149 (*ndhB*-C149). RNA-immunoprecipitation analysis indicated that OTP970 was associated with the *ndhB*-C149 site. The complementation of the *otp970* mutant with OTP970 lacking the DYW domain (OTP970^∆DYW^) failed to restore the RNA editing of *ndhB*-C149. *ndhB* gene encodes the B subunit of the NADH dehydrogenase-like (NDH) complex; however, neither NDH activity and stability nor NDH-PSI supercomplex formation were affected in *otp970* mutant compared to the wild type, indicating that alteration in amino acid sequence is not necessary for NdhB function. Together, these results suggest that OTP970 is involved in the RNA editing of *ndhB*-C149 and that the DYW domain is essential for its function.

## 1. Introduction

RNA editing is considered an indirect repair strategy that is required for the functional expression of organelle genomes [1]. RNA editing was first discovered in trypanosomes, where four Us were inserted into the mature mRNA of the cytochrome c oxidase II (coxII) in mitochondria (kinetoplasts), thus restoring the original function of the protein [2]. Subsequently, cytidine (C)-to-uridine (U) RNA editing in apolipoprotein-B48 mRNA in human and rabbit intestines [3] and adenine (A)-to-inosine (I) RNA editing in pre-mRNAs in other animals have been reported [4]. In terms of the conversion of C to U, the protein product of mature RNA following RNA-editing is different from that encoded by the genomic DNA, which may be attributed to the fact that C residues undergoing RNA editing are predominantly located at the first or second positions of codons. It is usually possible for C-to-U RNA editing to generate a new start codon by altering ACG to AUG [5] or to introduce a termination codon by altering CGA/CAA to UGA/UAA [6], thus extending or shortening the open reading frames. 

In the plant kingdom, C-to-U RNA editing was first discovered in mitochondria [7,8]. Two years later, the same type of RNAA editing was identified in chloroplasts [5]. In contrast, RNA editing has not been reported in the cytoplasmic RNAs of plants. Organelle RNA editing has been documented in almost all land plants, including bryophytes, ferns, gymnosperms, and angiosperms [9,10,11,12]. RNA editing usually occurs in the coding regions of mRNAs and occasionally in the non-coding regions of mRNAs, such as in the introns, and in transfer RNAs (tRNAs) [13,14,15]. 

In *Arabidopsis*, more than 40 and more than 600 C-to-U editing sites have been detected in RNAs transcribed from the chloroplast and mitochondrial genomes, respectively [16,17]. In vivo, in vitro, and organelle studies indicate that the nucleotide that is targeted for editing is recognized via 20–25 nt *cis*-elements located upstream (5′) of the editing site [18,19,20]. To date, a large number of *trans*-factors have been identified in the RNA editing process in plastids, including those involved in the editing of *ndhB* transcripts; the *ndhB* gene encodes the B subunit of the NADH dehydrogenase-like (NDH) complex that is involved in cyclic electron transport (CET) around the PSI. For example, CHLORORESPIRATORY REDUCTION 28 (CRR28), CRR22, EDITING LACKING INSERTIONAL MUTANT 1 (ELI1), ORGANELLE TRANSCRIPT PROCESSING 82 (OTP82), QUINTUPLE EDITING FACTOR 1 (QED1), CHLOROPLAST RNA EDITING FACTOR 7 (CREF7), and OTP84 are required for *ndhB* editing at sites 467, 746, 830, 836, 872, 1255, and 1481, respectively [21,22,23,24,25,26]. However, the *trans*-factors that are specifically required for the RNA editing of *ndhB* site 149 have not yet been identified. 

Pentatricopeptide repeat (PPR) proteins comprise an extraordinarily large protein family in land plants [27], with 450 members belonging to *Arabidopsis* and 477 in rice (*Oryza sativa*) [28]. The PPR proteins are defined by a tandem array (2 to 27 repeats) of the PPR motif, each of which contains 35 degenerate amino acids [29]. The PPR family members can be divided into the P and PLS subfamilies; the P proteins are composed of P motifs only, whereas the PLS proteins comprise the triplets of P-L-S motifs. A large number of recent studies show that PLS proteins are mainly involved in RNA editing. The PLS proteins are further divided into the PLS, E, and DYW subfamilies according to the differences in their amino acid sequence at the C-terminus [30]. Analysis of the crystal structure of these proteins reveals that each PPR motif folds into a pair of antiparallel α helices, with tandem arrays of PPR motifs forming a superhelix, which is the RNA-binding face [31,32,33]. The E motif is involved in protein–protein interactions [34,35], and the DYW domain contains a conserved zinc-binding motif (HxE(x)nPCxxC) that is similar to the cytidine deaminase domains, which have recently been shown to be able to crystallize, and its cytidine interim deaminase activity has been clearly proven [26,36,37]. 

The body of work described above shows that PLS proteins are crucial for RNA editing. However, the functions of most PPR proteins belonging to the PLS subfamily remain unidentified. In this study, we characterized the role of the OTP970 protein in the RNA editing of *ndhB*; An *otp970* T-DNA insertion mutant in *Arabidopsis* did not accumulate OTP970 and exhibited defective RNA editing at the *ndhB*-C419 site. Additionally, the OTP970 protein was found to bind to a *cis*-element surrounding *ndhB*-C149 in vivo. 

## 2. Materials and Methods

### 2.1. Plant Material and Growth Conditions 

The *Arabidopsis* ecotype Columbia (Col-0) was used as the wild type in this study. Seeds were sown on half-strength Murashige and Skoog (MS) medium containing 2% sucrose and were allowed to synchronize their germination at 4 °C for 3 days. Plants were grown at 22 °C under a 12 h light/12 h dark cycle. The *otp970* mutant (SALK_150217) was obtained from the *Arabidopsis* Biological Resource Center, OH, USA. The T-DNA insertion site in the *otp970* mutant was identified via the PCR amplification of genomic DNA and T-DNA borders using gene-specific primers (Table S1). 

### 2.2. RNA Isolation, RT-PCR, and RT-Quantitative PCR(QPCR)

Total RNA was extracted from the *otp970* mutant and wild-type plants using the Plant RNA Kit (Magen), and 5 μg total RNA was used for first strand cDNA synthesis, which was conducted using the PrimeScript RT Reagent Kit (TaKaRa). For the RNA editing analyses, 34 published RNA editing sites were amplified and sequenced using specific primers that spanned the editing site [38]. The level of RNA editing was calculated by comparing the relative heights of the nucleotide peaks at the editing site. For RT-PCR, *otp970* was amplified using gene-specific primers. RT-qPCR was carried out using the SYBR Premix Ex Taq™ (TaKaRa) on a real-time RT-PCR System (LC480; Roche). *Actin 2* was used as a reference gene. The primers used for RNA editing, RT-PCR, and RT-qPCR analyses are listed in Table S1. 

### 2.3. RNA Immunoprecipitation (RIP)

RIP assay was performed using 7-day-old *35S:OTP970-FLAG* transgenic and wild-type plants, as previously described [39]. Primers used in the analysis are listed in Table S1.

### 2.4. Thylakoid Membrane Preparation, Blue Native (BN)-PAGE, and Immunodetection

Thylakoid membranes were prepared as previously reported [40] and were quantified on the basis of their total chlorophyll content, also as previously described [41]. BN-PAGE was performed as previous report [42]. For immunodetection, thylakoid membrane proteins were separated on 12% SDS-urea-polyacrylamide gels, transferred onto polyvinylidene difluoride membranes (Millipore), and probed with specific primary antibodies purchased from Agrisera; the product numbers of the antisera were NdhB-AS16 4064, Cyt*f*-AS08 306, PsaB-AS10 695, PsbO-AS06 142-33, and ATPB-AS05 085. Signals were detected using the enhanced chemiluminescence method. 

### 2.5. Chlorophyll Fluorescence Analysis 

Chlorophyll fluorescence was detected with the MAXI version of the Imaging-PAMM–Series chlorophyll fluorescence system. Before measurement, plants were adapted to the dark for 30 min, and light–response curves were then measured as previously reported [42]. Transient increases in chlorophyll fluorescence were measured with dural-PAM 2000 after turning off the actinic light (AL), as previously described [43].

### 2.6. Complementation of otp970 Mutation

The wild-type At1g18485 genomic fragment (2910 bp) was PCR-amplified using gene-specific primers (Table S1) and was cloned into the pCAMBIA1301 binary vector. Transgenic lines were screened on half-strength Murashige and Skoog agar plates supplemented with 50 μg/mL hygromycin. Plants resistant to hygromycin were transferred to the soil to produce seeds. The complementation of *otp970* mutation was further confirmed by immunoblot analyses and by sequencing the editing site. 

### 2.7. OTP970 Protein Subcellular Localization

The OTP970 protein was fused with the green fluorescent protein (GFP) as previously described [44] but with slight modifications. A fragment encoding the first 100 N-terminal amino acids of OTP970 was amplified by RT-PCR using the primers listed in Table S1, and the fragment was cloned into the pUC18 under the control of 35S promoter to construct a fusion protein with GFP. 

## 3. Results 

### 3.1. RNA Editing at the ndhB-C149 Site Is Impaired in otp970 Mutant Plant

**Figure 1:**
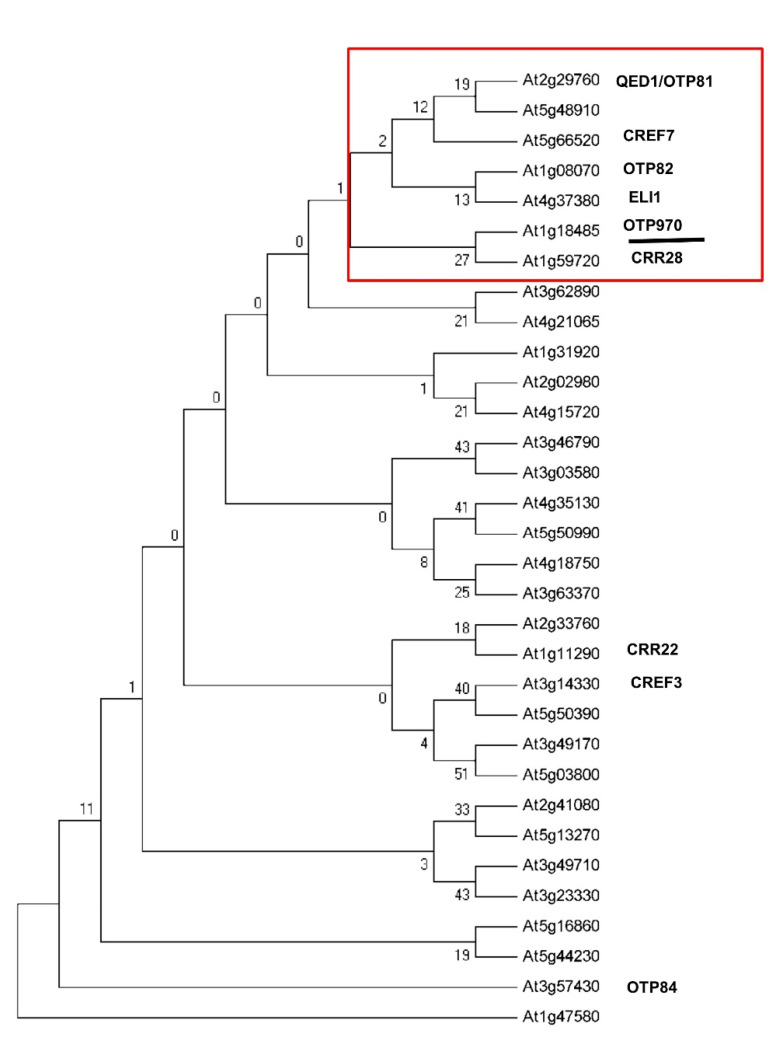
Phylogenetic analysis of the PLS-DYW subfamily of PPR proteins. In total, 32 *Arabidopsis* chloroplast-targeted PLS-DYW PPR proteins were selected for phylogenetic analysis. The neighbor-joining method was used to construct an unrooted phylogenetic tree with MEGA5. A co-evolution cluster enriched for *ndhB* RNA editing PPR proteins is outlined in red rectangles. The newly identified PPR Protein involved in RNA editing, OTP970 (At1g18485), is underlined.

**Figure 2:**
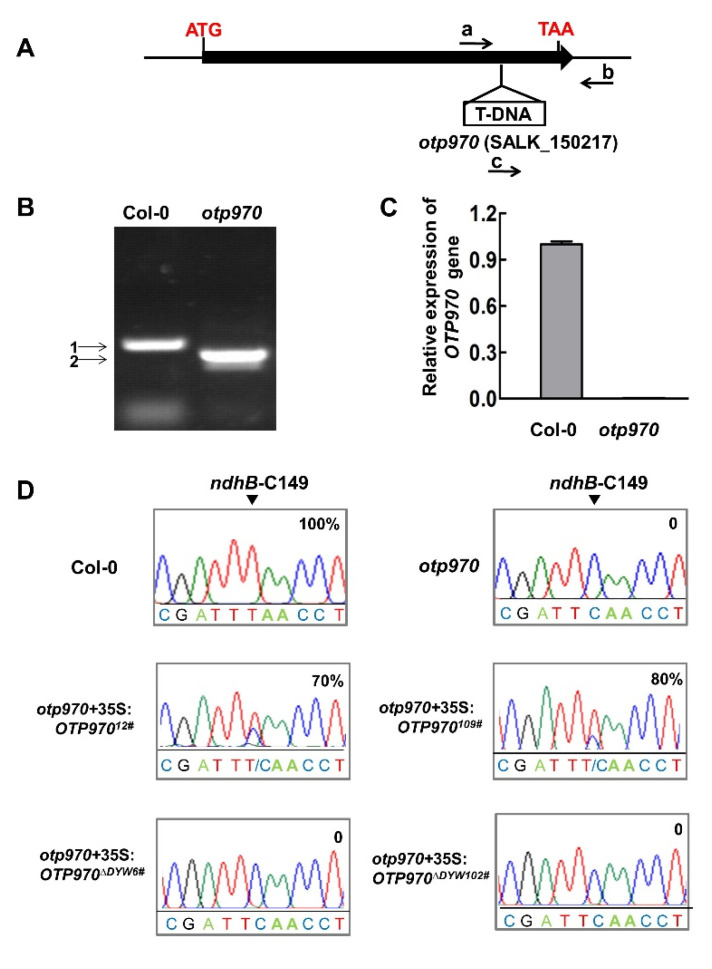
Molecular characterization of the role of OTP970 in RNA editing at *ndhB*-C149. (**A**) Schematic showing the T-DNA insertion site in the *otp970 Arabidopsis* mutant. Black rectangle and line represent the exon and UTR of the *OTP970* gene, respectively. ATG, start codon; TAA, stop codon. a, b and c represent primers SALK_150217 F, SALK_150217 R and Lbb1.3, respectively. (**B**) PCR amplification of *OTP970* genomic DNA from the wild type (Col-0) and *otp970* mutant to confirm the homozygosity of the mutant. 1 and 2 were amplified by using primers a, b and c shown in (**A**). (**C**) RT-qPCR analysis of *OTP970* transcription in the wild type and *otp970* mutant. Data represent the mean ± SE (*n* = 3). (**D**) Sequencing chromatograms of the RT-PCR products amplified from 3–4-week-old wild-type and mutant plants containing the *ndhB*-C149 editing site (arrow). *otp970 + 35S:OTP970^12#^* and *otp970 + 35S:OTP970^109#^*, *otp970* mutant transformed with wild-type *OTP970*; *otp970 + 35S:OTP970^∆DYW6#^* and *otp970 + 35S:OTP970^∆DYW102#^*, *otp970* mutant transformed with *OTP970* lacking the DYW motif. The editing efficiency is presented on the right side.

Chlorophyll fluorescence changes are often observed after plants have experienced AL illumination, which is indicative of NDH activity. Therefore, we used AL illumination to identify the mutants involved in NDH activity. However, this method cannot identify all of the possible NDH RNA editing mutants, as it may or may not affect NDH activity. The PLS subfamily members of the PPR proteins function in terms of the site recognition of the RNA editing. The DYW domain has been a strong candidate for the C deaminase activity required for C-to-U conversion in RNA editing [26]. To identify the additional PPR proteins that are involved in the RNA editing of *ndhB* in the chloroplasts, we focused on the phylogenetic analyses of the PPR proteins that belong to the PLS-DYW subfamily, which are predicted to be targeted to the plastids because previous research studies showed that the trans-factors involved in *ndhB* RNA editing all belong to the PLS-DYW subfamily. Because functionally related genes are believed to undergo co-evolution, we analyzed the co-evolution of the genes encoding PLS-DYW PPR proteins in *Arabidopsis* by means of hierarchical clustering. The results showed that several of the PPR genes that are involved in *ndhB* RNA editing were clustered together (Figure 1). We focused on a single co-evolution cluster comprising the following PPR proteins found to be involved in *ndhB* RNA editing: QED1/OTP81 (AT2g29760), CREF7 (At5g66520), OTP82 (At1g08070), ELI1 (At4g37380), and CRR28 (At1g59720). Among these, OTP970 was present, which is an intron-less gene that encodes a putative DYW PPR protein with 970 amino acids. OTP970 contains 23 characteristic PPR motifs, including PPR-related motifs in the E and DYW domains, as predicted by a web portal (https://ppr.plantenergy.uwa.edu.au/ accessed on 12 December 2021). We obtained an *otp970* mutant, the T-DNA was inserted in the coding sequence of At1g18485 (Figure 2A), and the T-DNA insertion site in the *otp970* mutant (Salk_150217) was confirmed by PCR (Figure 2B); We detected no *OTP970* transcripts in *otp970* homozygous mutant plants, as determined by RT-qPCR (Figure 2C); However, we observed no visible phenotypic differences between the mutant and wild-type plants. 

Several PPR proteins belonging to the PLS subfamily are involved in RNA editing in the organelles of land plants. To determine whether RNA editing was impaired in *otp970* mutant, we detected the editing status of the chloroplast transcripts. The RT-PCR products encompassing the 34 editing sites present in the *Arabidopsis* chloroplast transcripts [45] were directly sequenced. The results showed defective RNA editing at the *ndhB*-C149 site in the *otp970* mutant; this site was completely edited in wild-type plants but not in *otp970* mutants (Figure 2D). However, other RNA editing sites were not altered in transcripts from the *otp970* mutant. To further confirm that the defect in RNA editing at the *ndhB*-C149 site in the *otp970* mutant was due to the disruption of At1g18485, complementation was conducted by expressing OTP970 from the 35S promoter. Two complemented lines showed the 70% and 80% restoration of RNA editing at the *ndhB*-C149 site compared to the wild type, respectively (Figure 2D). Protein expression of the complemented lines were detected by immunoblot (Figure S1). Thus, At1g18485 successfully complemented the defective RNA-editing phenotype of the *otp970* mutant, suggesting that At1g18485 is the *OTP970* gene. 

Aberrant RNA processing can be a secondary cause of defective RNA editing [46]. To determine whether the defect in the RNA editing of *ndhB* was due to abnormal intron splicing, we performed RT-qPCR to examine the expression of *ndhB* in the *otp970* mutant. The abundance of *ndhB* transcripts was similar in the mutant and wild type (Figure S2A). Next, we examined intron splicing in the wild-type and *otp970* mutant plants by RT-qPCR and RT-PCR. *ndhB* showed no difference in terms of intron-splicing efficiency in the *otp970* mutant compared to in wild-type plants (Figure S2B,C). Taken together, our results indicated that the defective RNA editing of *ndhB* in the *otp970* mutant was not caused by aberrant intron splicing but was due to the loss of OTP970 function. 

### 3.2. Tissue-Specific Expression Pattern of OTP970

**Figure 3:**
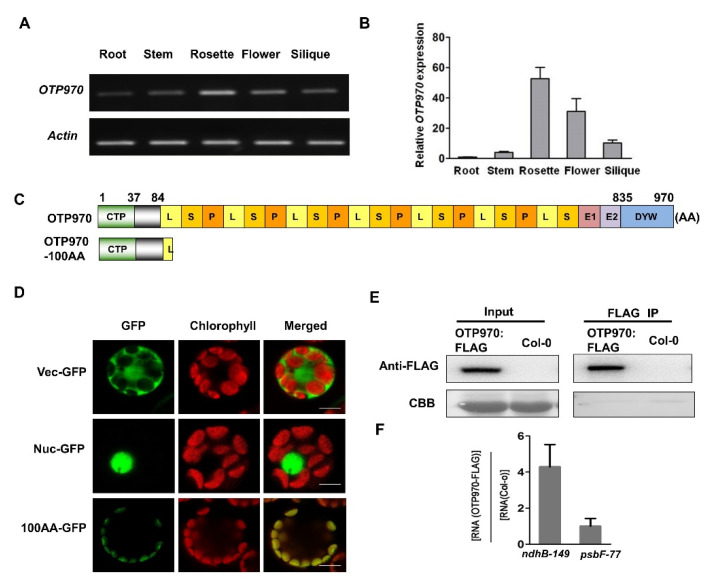
Expression pattern and subcellular localization of OTP970 and its association with *ndhB* at site 149 in vivo. (**A**) RT-PCR analysis of *OTP970* in root, stem, rosette, flower, and silique tissues of wild-type *Arabidopsis* (Col-0); *Actin 2* was used as a control gene. (**B**) RT-qPCR analysis of *OTP970* in root, stem, rosette, flower, and silique tissues of Col-0. Data represent the mean ± SE (*n* = 3). (**C**) Schematic representation of the GFP fusion constructs used in subcellular localization assays. A chimeric protein composed of the first 100 amino acids (putative chloroplast transit peptide (CTP) and a part of the mature OTP970) of At1g18485 is shown. (**D**) Localization of the OTP970 protein in the chloroplast. Vec-GFP, empty vector control; Nuc-GFP, nuclear control; 100AA-GFP, 1–100 N-terminal amino acids of OTP970 were fused to GFP. Scale bars = 10 μm. (**E**) Immunodetection of proteins extracted from wild-type (Col-0) and OTP970-FLAG transgenic plants. Proteins were immunoprecipitated (IP) using anti-FLAG antibody. CBB, Coomassie brilliant blue. (**F**) RT-qPCR analysis of the association of OTP970 with *ndhB* transcripts at site 149 and *psbF* transcripts at site 77 (negative control). Data represent the mean ± SE (*n* = 3).

Genevestigator was used to predict the expression of *OTP970*, and the expression data showed that *OTP970* mRNA is ubiquitously expressed in various *Arabidopsis* tissues [47]. To provide experimental evidence for the *OTP970* expression patterns, we extracted RNA from the root, stem, rosette, flower, and silique of a wild-type *Arabidopsis* plant and detected the expression of *OTP970* by RT-PCR and RT-qPCR with gene-specific primers. The results of RT-PCR analysis showed that *OTP970* was expressed in all vegetative and reproductive tissues (Figure 3A). Moreover, RT-qPCR data showed that the expression of *OTP970* was relatively highest in the rosette, intermediate in the flower, silique, and stem, and the lowest in the root (Figure 3B).

### 3.3. OTP970 Is Localized to Plastids and Associates with ndhB Transcripts at Site 149

According to the prediction of TargetP 1.1, 37 amino acids at the N-terminus of OTP970 are targeted to chloroplasts [48]. To validate this prediction experimentally, a fragment spanning the 1–100 N-terminal amino acids of OTP970 was fused to GFP (100AA-GFP) under the control of the Cauliflower Mosaic Virus *35S* promoter (Figure 3C), and the 100 AA-GFP fusion protein was transiently expressed in the *Arabidopsis* protoplasts. Analysis of the subcellular localization of the 100 AA-GFP fusion proteins by confocal laser scanning microscopy revealed that OTP970 is localized to the chloroplast (Figure 3D), which is consistent with the inference that OTP970 is involved in the RNA editing of plastid transcripts. We showed that OTP970 was involved in RNA editing at the *ndhB*-C149 site. To detect whether OTP970 is associated with these transcripts, we performed the RIP assay. The protein extracts of OTP970-FLAG transgenic and wild-type plants were immunoprecipitated using agarose beads coated with anti-FLAG antibody. Our results showed that OTP970-FLAG is enriched in the editing site of the *ndhB* transcript, while the enrichment of OTP970-FLAG at the *psbF* editing site is not apparent (Figure 3E,F). These results suggest that OTP970 is directly associated with the *ndhB* transcripts at site 149, which is consistent with a previous prediction [49].

### 3.4. DYW Motif of OTP970 Is Essential for RNA Editing

The DYW motif is suggested to be responsible for RNA editing, and its cytidine interim deaminase activity has been clearly proven [26,36,37]. To identify the role of the DYW motif of OTP970 in the *otp970* mutant, we expressed the OTP970 lacking the DYW motif (OTP970^∆DYW^) fused with two tandem FLAG tags in the *otp970* mutant under the control of the 35S promoter. The RNA editing efficiency at the *ndhB*-C149 site in the transgenic plants was similar to that in the *otp970* mutant (Figure 2D). Meanwhile, we detected the protein expression of complemented lines (Figure S3). These data suggest that the DYW motif of OTP970 is essential for RNA editing. 

### 3.5. NDH Function Is Not Impaired in otp970 Mutant Plant

**Figure 4:**
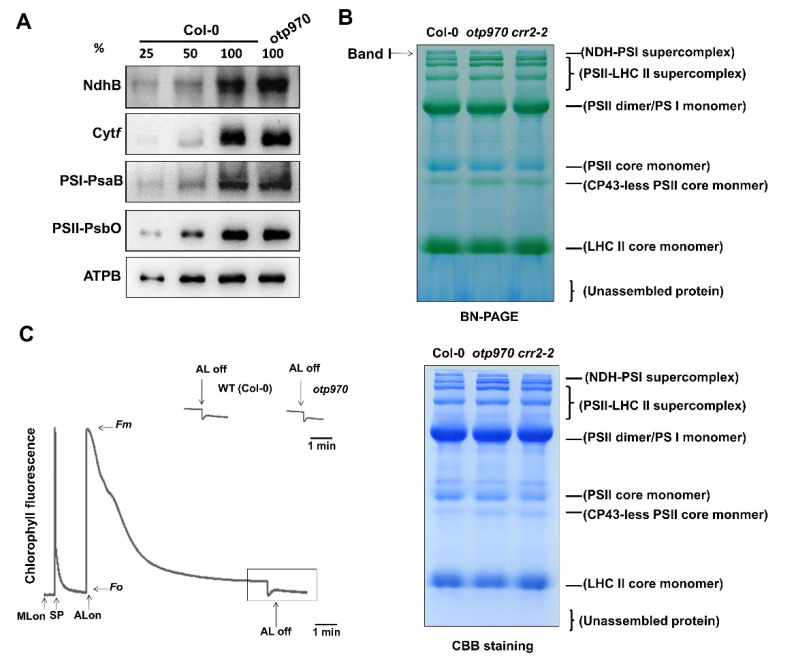
Analyses of the NAD(P)H dehydrogenase (NDH) complex in *otp970*. (**A**) Analysis of the NDH complex and the major photosynthetic complexes. Thylakoid membrane proteins of wild-type (Col-0) and *otp970* mutant plants were immunodetected with antisera raised against thylakoid protein. The antibodies against photosynthetic proteins were purchased from Agrisera. Total thylakoid protein samples containing equal amounts of chlorophyll were loaded in each lane. NdhB, a subunit of NDH; Cyt*f*, a subunit of the Cyt*b_6_f* complex; PsaB, a subunit of PSI; PsbO, a subunit of PSII; ATPB, a subunit of the ATP synthase complex. (**B**) BN-PAGE analysis of thylakoid proteins in Col-0, *otp970*, and *crr2-2*. Thylakoid membrane proteins in 10 μg of chlorophyll were loaded in each lane and were stained with Coomassie brilliant blue (CBB). NDH-PSI super complex (band I) is indicated with an arrow. PSI-NDH supercomplex-less mutant (*crr2-2*) was used for contrast. (**C**) NDH activity analysis by chlorophyll fluorescence measurements. Leaves were exposed to actinic light (AL) (50 μmol photons m^−2^ s^−1^) for 5 min, after which AL was turned off, and changes in the chlorophyll fluorescence levels were detected. Platquinone reduction based on NDH activity determined the transient increase in chlorophyll fluorescence. Insets are magnified traces from the boxed area. Fluorescence levels were normalized relative to the maximum fluorescence (F_m_) at closed PSII centers in the dark. ML, measuring light; SP, a saturating pulse of white light.

RNA editing at the *ndhB*-C149 site results in a Ser-to-Leu substitution at amino acid position 50. Therefore, a defect in RNA editing at the *ndhB*-C149 site in the *otp970* mutant may change the amino acid and may destabilize the NdhB protein in vivo. To detect this possibility, protein blots were immunodetected using the anti-NdhB antibody. The NDH complex consists of at least 29 subunits and may be destabilized by the loss of either NdhB or NdhD [50]. The NdhB protein level in the *otp970* mutant was similar to that in the wild type (Figure 4A), indicating that NdhB protein accumulation was not affected in *otp970*. Because RNA editing at the *ndhB*-C149 site in the *otp970* mutant was below the detection limit (Figure 2D), it is possible that the NdhB protein in the *otp970* mutant was translated from unedited RNA. This pattern is similar to other *trans*-factors, such as CRR22, which is involved in RNA editing at the *ndhB*-C746 and *ndhD*-C887 sites; CRR28, which is involved in RNA editing at the *ndhB*-C467 and *ndhD*-C878 sites; and CRR21, which is involved in RNA editing at the *ndhD*-C383 site [21,35]. Thus, we conclude that Ser-50 in NdhB (ndhB-50) is not essential for stabilizing the NDH complex. 

We also determined the accumulation of PsaB, Cyt*f*, PsbO, and ATPB, which are representative subunits of PSI, cytochrome (Cyt) *b_6_f* complex, PSII, and chloroplast F_0_F_1_-ATPase, respectively. The results showed that all of these photosynthetic complexes were not affected in the *otp970* mutant (Figure 4A). Consistently, there are no obvious defects were observed during photosynthetic electron transport in *otp970* (Figure S4). To assess whether RNA editing in *otp970* destabilizes the NDH-PSI supercomplex, BN-PAGE was used to analyze the accumulation of this supercomplex, and the *crr2-2* mutant was used as a PSI-NDH supercomplex-less control [50]. The abundance of the photosystem complexes corresponding to the NDH-PSI supercomplex in the *opt970* mutant was similar to that in the wild type but was reduced compared to the *crr2-2* mutant (Figure 4B, band I). This result suggests that the Ser-50 of NdhB is dispensable for the interaction between the NDH and PSI complexes.

Although CRR22, CRR28, and CRR21 are involved in the alteration of NDH activity, these proteins are not essential for stabilizing the NDH-PSI supercomplex and NDH complex [21,35]. To determine whether the function of OTP970 was similar to that of these CRR proteins, we analyzed the NDH activity in *opt970* by detecting the transient increase in chlorophyll fluorescence after turning off AL. The results showed no differences in chlorophyll fluorescence and post-illumination between the wild type and *otp970* mutant (Figure 4C), demonstrating that NDH activity was not affected in *otp970*. Taken together, these data suggest that the conversion of Ser-50 to Leu-50 in NdhB is not essential for the function of the NDH complex.

### 3.6. The Extent of RNA Editing at the ndhB-C149 Site Varies among Tissues 

**Figure 5:**
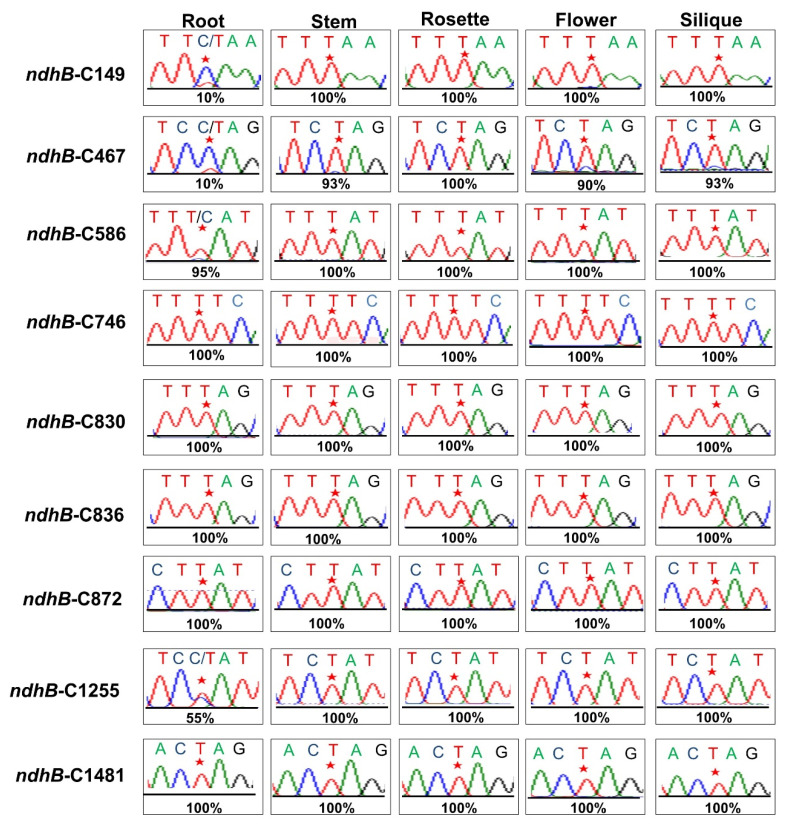
Analysis of the extent of RNA editing at nine *ndhB* sites in different tissues. RT-PCR products amplified from different tissues of wild-type (Col-0) were directly sequenced. All nine *ndhB* editing sites are indicated with asterisks above the corresponding peaks.

The extent of RNA editing varies among different tissues during plant development [51,52]. To detect whether the RNA editing that takes place at the *ndhB*-C149 site varied among different tissues, we conducted RT-PCR on the root, stem, rosettes, flower, and silique of wild-type plants during the reproductive phase. The RT-PCR sequencing products showed that RNA editing at the *ndhB*-C149 site was dramatically reduced in the root compared to in other tissues (Figure 5). Moreover, the rate of RNA editing at *ndhB*-C467 and *ndhB*-C1255 sites were also dramatically reduced in the root, whereas that at the *ndhB*-C586 site showed a slight reduction in the root compared to other tissues (Figure 5). 

## 4. Discussion

RNA editing is an important post-transcriptional regulation in plastids. Although some regulators have been identified in the RNA editing process in plastids [53], there are still regulators that are specifically required at some RNA editing sites in the plastids that have not yet been identified. In this study, we identified and characterized the role of OTP970 encoding a PLS-DYW subfamily of PPR proteins in RNA editing at the *ndhB*-C149 site that has not been identified in *Arabidopsis* plastids. 

The chloroplast NDH complex is divided into four subcomplexes, including the membrane, lumen, and stroma-exposed A and B subcomplexes, based on the characterization of the eubacterial NDH complex and *Arabidopsis* mutants lacking NDH subunits [54]. The membrane subcomplex contains seven plastid-encoded subunits (NdhA–NdhG), whereas the stroma-exposed A subcomplex includes four plastid-encoded subunits (NdhH–NdhK) and four nuclear-encoded subunits (NdhM–NdhO) [54]. The activity and stability of the NDH complex and its ability to form a supercomplex with PSI are impaired in mutants lacking the proteins required for the assembly of the stroma-exposed subcomplexes [55,56]. 

The C-to-U editing of the *ndh* transcripts alters the amino acid sequence of the encoded subunit proteins at certain sites; thus, the functional importance of amino acids varies with site changes. For example, defects in RNA editing at the *ndhB*-C746 and *ndhD*-C887 sites in *crr22*, *ndhB*-C467, and *ndhD*-C878 sites in *crr28* and at the *ndhD*-C383 site in *crr21* can impair NDH activity [21,35]; however, these alterations do not affect the stability of the NDH complex. In this study, we also observed that the *otp970* mutant was defective in RNA editing at *ndhB*-C149; nevertheless, NDH activity and stability of the NDH complex were unaffected compared to in the wild type (Figure 4). This is similar to the *otp82* mutant, in which the RNA editing defects at the *ndhB*-C836 and *ndhG*-C50 sites did not affect NDH function [23]. 

NDH-dependent CET plays a crucial role in response to various stresses. For example, tobacco mutant NDH complex defects show no differences in photosynthetic activity when compared to wild-type [43,57]. However, NDH-defective mutants are sensitive to various stress, such as high light intensity [58], low humidity [57], drought [59], and high/low temperature [60,61], indicating that the NDH complex protects the photosynthetic activity in the chloroplasts from oxidative stress. Thus, these editing sites that do not affect NDH function under normal conditions but probably play important roles under certain stress conditions, or mediate NDH function maintenance during certain evolutionary periods. 

Previous studies have shown that RNA editing at individual sites occurs in some but not all species [62]. Additionally, the extent at which RNA editing occurs at the same site varies across different developmental stages and/or among different tissues [51,52]. In this study, our results showed that the degree of C-to-U editing at the *ndhB*-C149 site was 100% in the stem, rosette, flower, and silique tissues, but only ~10% in the root sample (Figure 5). Notably, the *ndhB*-149 in the stem is fully edited, but the expression patterns indicated that *OTP970* was expressed at very low levels in stems (Figure 3A), implying that differences in editing rates among these tissues probably resulted from both the expression and binding activity of their corresponding *trans*-acting factors. 

## 5. Conclusions

In this paper, we identified *OTP970*, a gene encoding the PLS-type PPR protein, which is required for RNA editing in plastids. The sequencing results of RT-PCR products showed that *otp970* mutant was impaired in RNA editing at the *ndhB*-C149 site. RIP analysis demonstrated that OTP970 was associated with *ndhB*-C149 site. Furthermore, we discovered that the DYW domain of OTP970 is required for RNA editing at the *ndhB*-C149 site. These findings increased our understanding of the mechanism in plastid RNA editing.

## Data Availability

Not applicable.

## Supplementary Materials

The following supporting information can be downloaded at: https://www.mdpi.com/article/10.3390/genes13010139/s1, Figure S1: Western blot analysis of *otp970* complemented *35:OTP970/otp970* plants, Figure S2: Transcript profiles of genes with the editing defects in *otp970* mutant, Figure S3: Western blot analysis of the OTP970 lacking the DYW motif (OTP970^∆DYW^), Figure S4: In vivo analysis of electron transport activity in wild-type and *otp970 Arabidopsis* mutant plants, Table S1: List of primers used in this study.
