# Supplementary material for: OTP970 Is Required for RNA Editing of Chloroplast ndhB Transcripts in Arabidopsis thaliana
Source: Genes (Basel). 2022 Jan 14;13(1):139. doi: 10.3390/genes13010139 (PMC8774829; doi:10.3390/genes13010139)
Supplement: Supplementary file 1 [file genes-13-00139-s001.zip › genes-1530327-supplementary.pdf]

Supplementary data

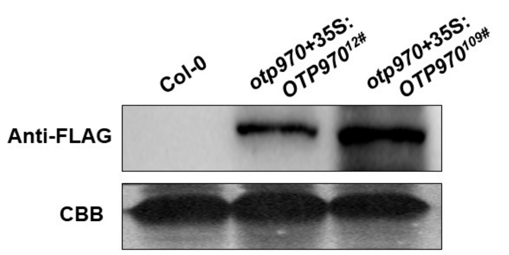

Figure S1. Western blot analysis of *otp970* complemented *35:OTP970/otp970* plants.

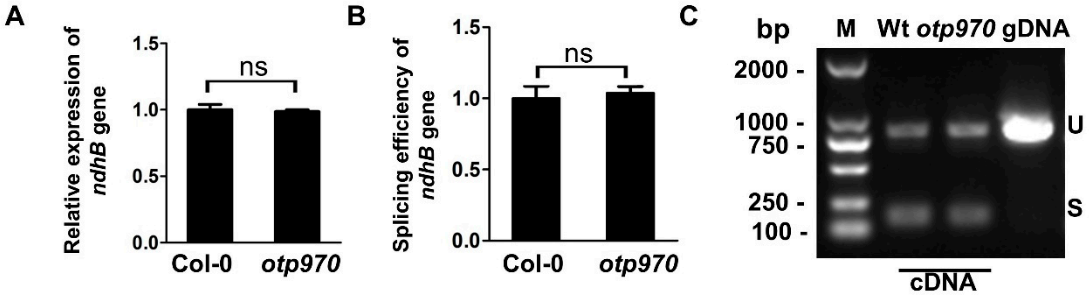

Figure S2. Transcript profiles of genes with the editing defects in *otp970*. (A) RT–qPCR-based analysis of *ndhB* expression. (B) RT–qPCR-based analysis of splicing efficiency of *ndhB* in wild-type and *otp970* plant. (C) RT–PCR analysis of the *ndhB* transcripts from wild-type (Col-0), *otp970* plant. Molecular weight markers (M) are shown on the left; the product amplified from total DNA (gDNA) is shown in the right side. S, Spliced; U, unspliced. Significant differences were identified using the Student's *t*-test, ns indicated no significant differences.

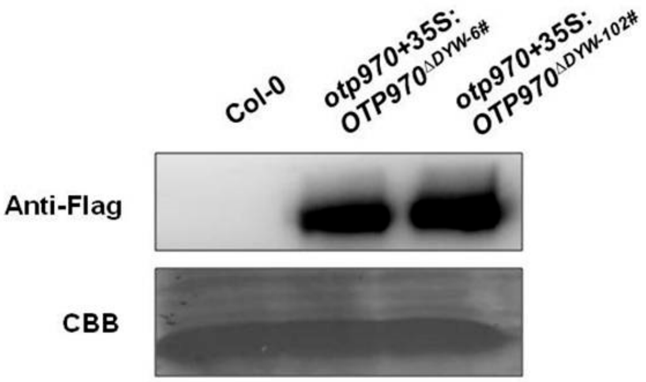

Figure S3. Western blot analysis of the OTP970 lacking the DYW motif (OTP970$^{\Delta DYW}$). OTP970$^{\Delta DYW}$ protein was fused with flag and then transformed in *otp970*. Anti-Flag antibody was used to detection the wild-type (Col-0) and two transgenic plants. CBB, Coomassie brilliant blue.

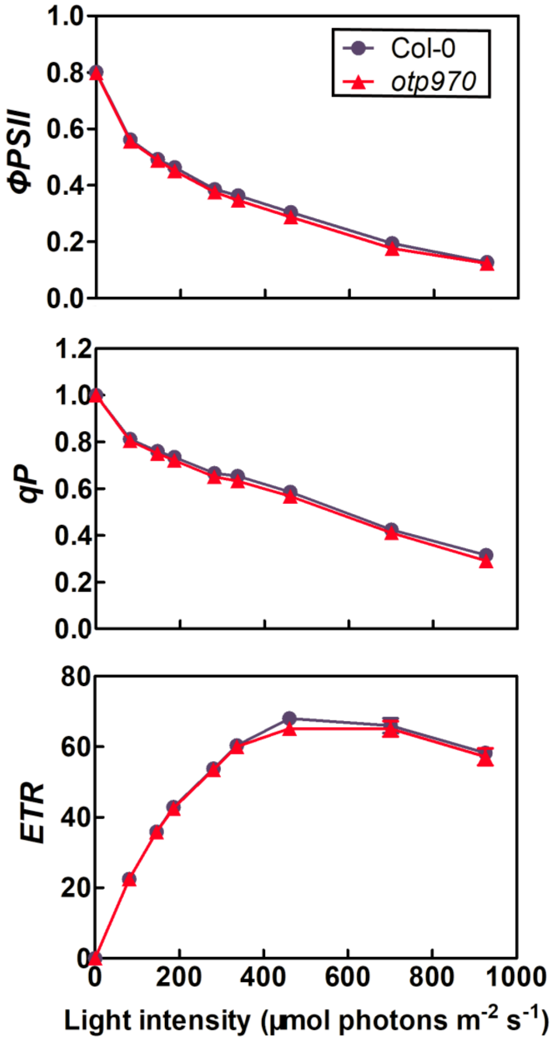

Figure S4. In vivo analysis of electron transport activity in wild-type and *otp970* Arabidopsis mutant plants. Light-response curves of PSII quantum yield (Φ_{PSII}), photochemical quenching (qP), and electron transport rate (ETR) from 4-week-old Col-0 and *otp970* plants. Chl fluorescence measurements were performed at the following light intensities: 0, 81, 145, 186, 281, 335, 461, 701 and 926 μmol photons m^{-2} s^{-1}. Data for wild-type and *otp970* plants are presented as the mean ± SE of triplicates.

Table S1. List of primers used in this study

Primer	Sequence
Mutants Identification	
SALK_150217-F	5'- TGGTTTTGTATGGAATCCAGC-3'
SALK_150217-R	5'- AACATTGATGTTTGTCTCGCC-3'
Real Time-PCR	
OTP970-F	5'- AAGTTGCTGCTAAGTTATTCG-3'
OTP970-R	5'-CACTTAGATCATGTTGTACTGACA-3'
ndhB-F	5'- TCATCAATGGACTCCTGACG -3'
ndhB-R	5'- CCAGAAGAAGATGCCATTCA -3'
ndhB introns-F	5'- AGTCTCATGCACGGTTTTGA -3'
ndhB introns-R	5'- CCAGAAGAAGATGCCATTCA -3'
ACTIN 2-F	5'-GGTAACATTGTGCTCAGTGGTG-3'
ACTIN 2-R	5'-CTCGGCCTTGGAGATCCACATC-3'
GFP Assay	
OTP970-100AA-F	5'-CCTGGCGCGCCACTAGTGGATCCATGGCTTCTGTTTTGCTTCC-3'
OTP970-100AA-R	5'-GAGCGGTACCCTCGAGGTCGACAATGTCTTTTCTCTTCCCA-3'
RIP Assay	
ndhB-149-F	5'-CTTCTGATGATCGATTCAACC-3'
ndhB-149-R	5'-TCAATGTACTCTACGGATAGAGG-3'
psbF-77-F	5'-GGACCTATCCAATTTTTACAGTGC-3'
psbF-77-R	5'-GTTGGATGAACTGCATTGCT-3'
Complementation Assay	
OTP970cDNA-F	5'-CACGGGGGACTAAGCTTATGGCTTCTGTTTTGCTTCC-3'
OTP970cDNA-R	5'-CCTTGTAATCACTAGTCCAGTAATCTCCACAAGAAC-3'
OTP970^{DYW}cDNA-F	5'- CACGGGGGACTAAGCTTATGGCTTCTGTTTTGCTTCC-3'
OTP970^{DYW}cDNA-R	5'-CCTTGTAATCACTAGTCTTTCGTAAACTCATCTCGT-3'
RT-PCR Assay	

OTP970-F	5'- TGTCTGATGTTGGGATCGG -3'
OTP970-R	5'- CATGTGTATCACCCTCTGCTG-3'
ndhB-F	5'- TCATCAATGGACTCCTGACG -3'
ndhB-R	5'- CCAGAAGAAGATGCCATTCA -3'
ACTIN 2-F	5'- CTCTTCCTCATGCCATCCTC-3'
ACTIN 2-R	5'-GCTCATACGGTCAGCGATAC-3'
